# Multiple Regions of Kaposi’s Sarcoma-Associated Herpesvirus ORF59 RNA are Required for Its Expression Mediated by Viral ORF57 and Cellular RBM15

**DOI:** 10.3390/v7020496

**Published:** 2015-02-03

**Authors:** Maria Julia Massimelli, Vladimir Majerciak, Jeong-Gu Kang, David J. Liewehr, Seth M. Steinberg, Zhi-Ming Zheng

**Affiliations:** 1 Tumor Virus RNA Biology Section, Gene Regulation and Chromosome Biology Laboratory, Center for Cancer Research, National Cancer Institute, National Institutes of Health, 1050 Boyles Street, Frederick, MD 21702, USA; E-Mails: julia.massimelli@uci.edu (M.J.M.); majerciv@mail.nih.gov (V.M.); jkang@jkbiopharma.com (J.-G.K.); 2 Biostatistics & Data Management Section, Center for Cancer Research, National Cancer Institute, National Institutes of Health, Bethesda, MD 20892, USA; E-Mails: liewehrd@mail.nih.gov (D.J.L.); steinbes@mail.nih.gov (S.M.S.)

**Keywords:** KSHV, ORF59, ORF57, RNA accumulation, RNA export, RBM15

## Abstract

KSHV ORF57 (MTA) promotes RNA stability of ORF59, a viral DNA polymerase processivity factor. Here, we show that the integrity of both ORF59 RNA ends is necessary for ORF57-mediated ORF59 expression and deletion of both 5’ and 3’ regions, or one end region with a central region, of ORF59 RNA prevents ORF57-mediated translation of ORF59. The ORF59 sequence between nt 96633 and 96559 resembles other known MTA-responsive elements (MREs). ORF57 specifically binds to a stem-loop region from nt 96596–96572 of the MRE, which also binds cellular RBM15. Internal deletion of the MRE from ORF59 led to poor export, but accumulation of nuclear ORF59 RNA in the presence of ORF57 or RBM15. Despite of being translatable in the presence of ORF57, this deletion mutant exhibits translational defect in the presence of RBM15. Together, our results provide novel insight into the roles of ORF57 and RBM15 in ORF59 RNA accumulation and protein translation.

## 1. Introduction

Kaposi’s sarcoma-associated herpesvirus is the causative agent of multiple human malignancies, including Kaposi’s sarcoma (KS), primary effusion lymphomas (PELs), and multicentric Castleman’s disease (MCD). Following primary infection, KSHV establishes a lifelong latency in which the KSHV episome is replicated by the host cell replication machinery. Upon reactivation to lytic cycle, the replication of KSHV genome depends on several viral proteins, including DNA polymerase (Pol-8, ORF9), processivity factor of DNA polymerase (PF-8, ORF59), helicase (HEL, ORF44), primase (PRI, ORF56), primase-associated factor (ORF40/41), single-strand DNA binding protein (SSB, ORF6), replication and transcription activator (ORF50 or RTA), and replication-associated protein (RAP, K8) [1,2,3]. ORF59 is a phosphoprotein that binds dsDNA and the DNA polymerase Pol-8, promoting the synthesis of full-length DNA by acting as a sliding clamp [4]. ORF59 protein is phosphorylated by KSHV viral Ser/Thr kinase (ORF36) and this step seems to be critical for ORF59 activity and viral DNA synthesis [5]. RTA activates ORF59 transcription [6,7,8] and recruits ORF59 protein to the origin of lytic replication (*ori*Lyt) [3,4,9,10]. RTA also activates other early/delayed-early/late genes necessary for the successful production of infectious viral particles [6,7]. Lytic replication accelerates host mRNA turnover [11], but promotes stability of viral RNAs to ensure successful virus lytic replication. KSHV ORF57 is a viral early, dimerable protein [12]. ORF57 promotes accumulation of numerous viral transcripts, thereby naming mRNA transcript accumulation (MTA) [13,14,15,16], and has many other functions [17]. ORF57 binds to a structured sequence motif in the RNA, which is named as an MTA-responsive element (MRE), in association with cellular proteins to promote viral RNA stability [18,19,20]. We showed that ORF57 binds to ORF59 RNA [13,18] and promotes ORF59 RNA accumulation by interacting with cellular proteins RBM15 and OTT3 [15]. Direct interactions of ORF57 with RBM15 and OTT3 interferes with RBM15 or OTT3 binding to ORF59 RNA and prevents RBM15/OTT3-mediated nuclear accumulation and hyperpolyadenylation of ORF59 RNA [15]. Here, we present the further evidence of RNA cis-elements in ORF59 in regulation of ORF59 RNA export and protein translation.

## 2. Materials and Methods

### 2.1. The Expression Vectors

pVM7 for FLAG-tagged ORF57, pVM68 for 3 × FLAG-tagged ORF57, pVM18 for FLAG-tagged ORF59 and RBM15-FLAG were described in previous reports [13,21,22]. The mutant (mt) ORF59-FLAG expression vectors constructed in this study are listed in Table S1. The oligos used for plasmid construction are listed in Table S2. Overlapped PCR for construction of truncation mutants was performed as described [23] and all of truncation mutations were constructed in-frame with a FLAG-tag to express a truncated ORF59-FLAG fusion.

### 2.2. Cells and Co-Transfection Assays

Human HEK293 cells were cultivated in DMEM supplemented with 10% fetal bovine serum (FBS). All co-transfection assays were carried out in HEK293 cells (5 × 10^5^/well) plated in a six-well plate. Twenty four hrs after seeding the cells were co-transfected with 1 µg of individual ORF59 expression vector together with 0.2 µg of ORF57 (pVM7 or pVM68) or RBM15 vectors. The cells cotransfected with an empty pFLAG-CMV-5.1 vector were used as a negative control. All transfections were carried out with Lipofectamine 2000 (Invitrogen, Carlsbad, CA, USA) as recommended. Protein samples were obtained by direct cell lysis with 0.5 mL of 2× SDS protein loading solution (Quality Biological, Inc., Gathersburg, MD, USA) supplemented with 5% 2-mercaptoethanol and analyzed by Western blotting with following antibodies: rabbit polyclonal anti-ORF57 antibody [24] (1:3000), rabbit polyclonal anti-RBM15 antibody (Proteintech Group, Inc., Chicago, IL, USA), mouse monoclonal anti-ORF57 antibody (unpublished data, used at a dilution of 1:1000); monoclonal anti-FLAG M2 antibody (1:3000, Sigma, St. Louis, MO, USA, F1804); monoclonal anti-β-tubulin (1:3000, Sigma, T5201), together with corresponding peroxidase-conjugated secondary antibodies (1:10,000, Sigma). The signal on the Western blot was detected with SuperSignal West Pico Chemiluminiscence Substrate (Pierce, Rockford, IL, USA). Total cell RNA samples were prepared 24 or 48 h after transfection by the addition of 1 mL of TRIzol reagent (Life Technologies, Carlsbad, CA, USA). Extraction of cytoplasmic and nuclear total RNA and Northern blot analysis was performed as described before [13]. The expression level of gene of interest was determined by Northern blot analysis using the following γ-^32^P-labeled antisense oligo probes: oVM11 for ORF57, oVM158 and oJM65 for ORF59, oZMZ270 for GAPDH and oST197 for U6 small nuclear RNA (see Table S2 for each oligo sequence). The autoradiograph was captured using a Molecular Dynamics PhosphorImager Storm 860 and analyzed with ImageQuant software (GE Healthcare Bio-Sciences, Pistataway, NJ, USA).

### 2.3. RNA-Protein Pulldown Assays

KSHV-infected, engineered BCBL1 cells, TREx BCBL1-vector and TREx BCBL1-RTA cells [25], were cultivated in RPMI 1640 supplemented with 10% FBS and hygromycin B (50 µg/mL). To induce the expression of KSHV lytic genes, TREx BCBL1-RTA or TREx BCBL1-vector (a negative control) cells were treated with 1 µg/mL of doxycycline (Dox) for 24 h. Total cell extract was prepared from ~5 × 10^6^ of TREx BCBL1–RTA or –vector cells in RIPA buffer as described [19]. RNA-protein pull down assays were performed as described previously [18,26] using customized 5’-biotinylated RNA oligos oJM36, oJM37, oJM38, or oJM39 derived from ORF59 RNA (Table S2) and oNP42 or oNP41 derived from vIL6 (Table S2) [18]. The proteins associated with each RNA oligo were analyzed by Western blotting.

### 2.4. Quantitative RT-PCR and RNA Decay Analysis

ORF59 RNA decay was analyzed in HEK293 cells transiently transfected with an ORF59 expression vector in the presence or absence of ORF57 as described previously [15,19]. The following ORF59 TaqMan primers from IDT (Coralville, IA, USA) were used: ORF59 Probe, ORF59 primer 1 and ORF59 primer 2 (Table S2). The relative expression of ORF59 and non-linear regression analysis of the data were determined as described [15,19].

## 3. Results and Discussion

### 3.1. Mapping of the Regions in ORF59 RNA Responsible for ORF57-Mediated RNA Accumulation and Protein Translation

**Figure 1:**
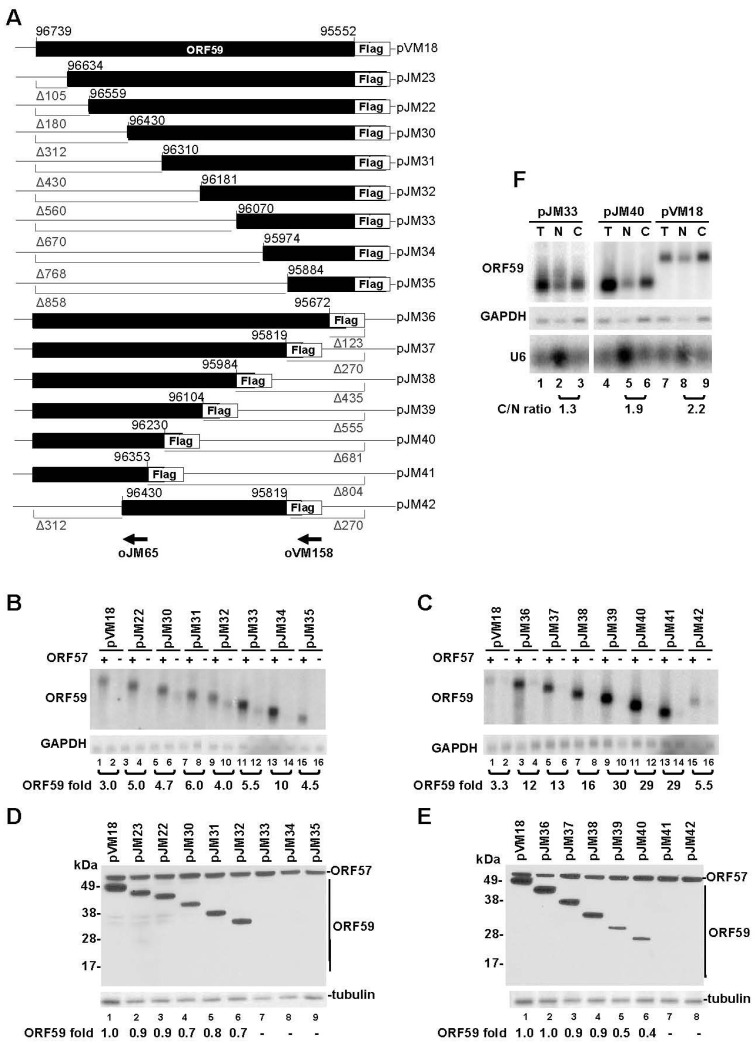
Mapping of ORF57-responsive elements in ORF59 RNA. (**A**) Diagrams of wt ORF59 and its progressive 5’ and 3’ deletion mutants fused in-frame with FLAG-tag on the C-terminus. The size (in nts) of each deletion (Δ) is indicated below the gray line. Numbers above each construct are nt positions in the KSHV genome (GenBank Ac. No. U75698.1). Arrows in the corresponding ORF59 positions are antisense oligomers (oVM158 or oJM65) used for detection of ORF59 RNA in panel (B) and (C) by Northern blot. (**B**–**E**) The RNA and protein expression of ORF59 wt and its deletion mutants in the presence or absence of ORF57. HEK293 cells were transfected with ORF59-FLAG expression vectors in the presence (+) or absence (−) of ORF57 and total RNA and proteins were isolated 24 h after transfection. Northern blot analysis of ORF59 expression with a γ-^32^P-labeled antisense oligo probe oVM158 or oJM65 for ORF59 RNA in panels B and C. The relative level of ORF59 in each sample was calculated after normalization to GAPDH, detected by a γ-^32^P-labeled antisense oligo oZMZ270, used as a loading control. A relative ratio (fold) of ORF59 in the presence over the absence of ORF57 was determined according to the normalized levels in each sample pair. Protein expression profiles of wt ORF59 and its in-frame deletion mutants in the presence of ORF57 are shown in panels (D) and (E). Protein samples from HEK293 cells with each construct transfection as described above in the presence of ORF57 were analyzed by Western blot with anti-FLAG for both ORF59 (wild type and mutants) and ORF57 expression or anti-β-tubulin for sample loading. The relative protein level showed at the bottom of each Western blot panel was calculated by measurement of protein signal intensity after normalization to β-tubulin, with the wt ORF59 protein level being set to 1. (**F**) RNA expressed from both wt ORF59 and its deletion mutants is exportable. Total (T) and fractionated nuclear (N) and cytoplasmic (C) RNA were isolated from HEK293 cells at 24 h of transfection with a wt ORF59 (pVM18) or its two deletion mutants (a 5’ deletion mt pJM33 and a 3’ deletion mutant pJM40) in the presence of ORF57 and were analyzed by Northern blot as described above. GAPDH RNA served as a control for sample loading and nuclear U6 RNA served as a fractionation efficiency control. The relative level of ORF59 RNA in the cytoplasm *versus* the nucleus for each expression vector was calculated as a C/N ratio.

In an attempt to delineate ORF59 sequences responsible for regulation by ORF57, we constructed a series of progressive 5’ to 3’ and 3’ to 5’ truncations of ORF59 (Figure 1A) and evaluated the relevance of these sequences for accumulation of ORF59 RNA and translation of ORF59 protein in the presence or absence of ORF57. Progressive truncations from the 5’- or 3’-end of ORF59 were found to gradually increase the accumulation of total ORF59 RNA in the presence of ORF57 (Figure 1B,C), in particular from the 3’-end truncation (Figure 1C), but appeared to gradually decrease the translation of ORF59 protein (Figure 1D,E). Surprisingly, three expression vectors pJM33-35 with the progressive 5’ to 3’ ORF59 truncation and an expression vector pJM41 with the 3’ to 5’ ORF59 truncation exhibited no protein translation in the presence of ORF57 (Figure 1D, E) despite they were constructed in-frame with a C-terminal FLAG tag. As the expression vectors pJM32 and pJM40 could translate ORF59, we initially assumed that the sequence motifs from nt 96181 to 96070 and from nt 96353 to 96230 in ORF59 RNA are important for ORF59 translation in response to ORF57, although the truncation from nt 95984 to 96104 already displayed a marked reduction of ORF59 translation (compare pJM38 to pJM39) (Figure 1D, E). Further analysis of a construct pJM42, which has both deletion of 312 nts from the 5’ end as seen in pJM30 and the deletion of 270 nts from the 3’ end as seen in pJM37, also showed the lack of ORF59 translation in the presence of ORF57, indicating that two cis-elements, each at the 5’ half or at the 3’ half of ORF59 are important for ORF59 translation in the presence of ORF57. Two representative mutants, pJM33 with 670 nt truncation at the 5’ end and pJM40 with 681 nt truncation at the 3’ end, were analyzed for their RNA export by cytoplasmic (C) and nuclear (N) fractionation (Figure 1F). Mutant pJM33 expressed no detectable ORF59 protein (Figure 1D, Lane 7) while mutant pJM40 had ~60% reduction of ORF59 protein expression compared to the wild type (Figure 1E, Lane 6). Although the pJM33 did exhibit a relative lower C/N ratio (1.3) compared to that of pJM40 and wild type RNA (1.9 and 2.2, respectively) (Figure 1F), both ORF59 mutants were able to export RNA to the cytoplasm (Figure 1F, Lanes 3 and 6). Therefore, the reduced or absent ORF59 protein expression observed in these truncation mutants in the presence of ORF57 could not be resulted from this small change of C/N ratio.

It has previously been shown that ORF57 is capable of enhancing translation of viral mRNAs by recruiting PYM, a cellular protein capable of facilitating the assembly of the 48S pre-initiation complex onto viral intronless mRNAs [27]. As all truncated mutants analyzed in Figure 1 show higher RNA accumulation in the presence of ORF57, we hypothesized that ORF57 promotion of ORF59 translation requires at least one RNA cis-element either at the 5’-end or at the 3’-ends of ORF59 mRNA to sustain its translational initiation activity for a certain size of ORF59 RNA, but lacking of both leads to the complete abolishment of its activity in initiation of ORF59 translation. Alternatively, a truncated ORF59 protein expressed from pJM33, pJM34, pJM35, pJM41, or pJM42 might be a labile protein because of lacking one (pJM33, pJM34, pJM35, and pJM41) or two (pJM42) dimerization domains on either ends of ORF59 protein [3]. Another uncharacterized domain encoded from nt 96181(pJM32)-96070 (pJM33) might be also required to stabilize the protein when one dimerization domain is missing from ORF59. In supporting this notion, we also observed that a remarkable reduction of the truncated ORF59 protein expression from pJM39 over that of pJM38, when further deletion was made from nt 95984 (pJM38) to 96104 (pJM39) (Figure 1E). Further deletion to nt 96353 (pJM41) makes this truncated protein undetectable. Deletion of the phosphorylation sites (Ser376, Ser378, and Ser379) on the C-terminus of ORF59 [5] does not appear to affect the protein stability.

### 3.2. The 5' MRE of ORF59 Interacts with ORF57 and RBM15 and Exhibits a Role in RNA Export and Stability

**Figure 2:**
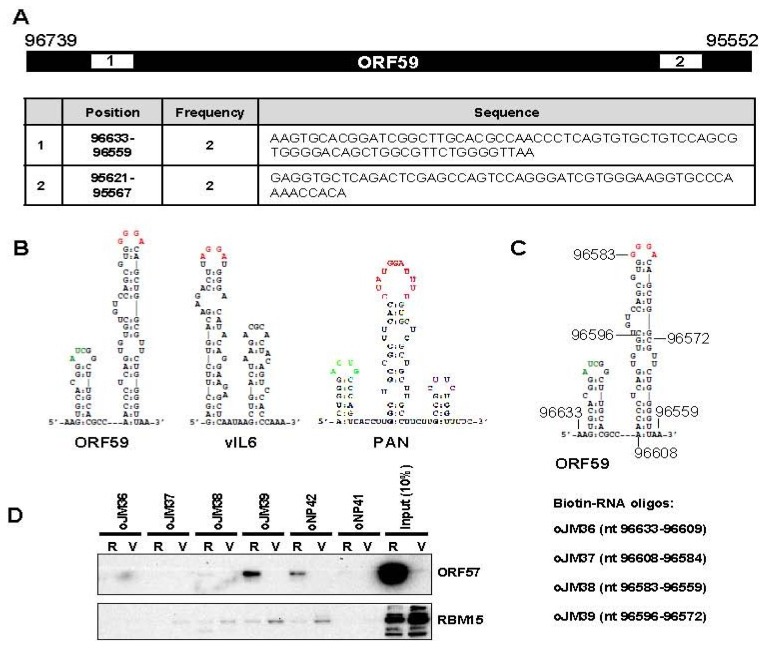
A MRE in the 5’ ORF59 interacts with ORF57 and RBM15. (**A**) Two ORF59 regions (numbered white boxes) identified in ORF57 CLIP assay [18]. Below is the table showing their positions, cloning frequency and nucleotide sequence. (**B**) Secondary structures of the identified RNA sequences in ORF59, vIL6 and PAN RNA by anti-ORF57 CLIP. The nucleotide sequences in the stem-loop in red color are the interacting site for ORF57 and its associated cellular proteins. (**C**) The ORF59 5’ MRE structure with the nt positions of biotinylated RNA oligomers used in RNA pull-down assays. (**D**) KSHV ORF57 and cellular RBM15 preferentially bind to a stem-loop structure of the MRE. Cell lysates prepared from TREx BCBL1-RTA (R) or -vector (V) cells induced with Dox for 24 h were used for the RNA pulldown assays with an indicated RNA oligomer. The RNA oligo oNP42 derived from vIL6 RNA was used as a positive control and oNP41 oligo as negative control [18]. ORF57 or RBM15 associated with RNA oligos in the pulldowns was immunoblotted using an anti-ORF57 or anti-RBM15 antibody. The cell lysate (10%) prior to the pulldown served as an input control in Western blot.

Previous anti-ORF57 CLIP assays [18] identified two regions within ORF59 RNA interacting with ORF57 (Figure 2A), one at the 5’-end and the other at the 3’-end of ORF59 RNA. We selected the region at the 5’ end of ORF59 (nt 96633–96559) that shows a pronounced structure and sequence similarity to previously identified MTA-responsive elements (MREs) [19,20,28] (Figure 2B). We called this region as the ORF59 MRE. In order to provide direct biochemical evidence of the ORF59 MRE interaction with ORF57, we performed RNA-pull down assays using customized biotinylated RNA oligos covering the entire ORF59 MRE (oJM36, oJM37, oJM38, oJM39) as showed in Figure 2C. KSHV ORF57 binding to individual oligomer was tested in the cell extract prepared from KSHV-infected TREx BCBL1-RTA (R) cells expressing ORF57 after induction of RTA expression with DOX. The cell extract from TREx BCBL1-vector (V) cells without ORF57 expression were used for comparison. Western blot analysis of the pulled down proteins showed the binding of ORF57 protein to oJM39 RNA overlapping nt 96595–96572, a loop structure of ORF59 MRE. In fact, ORF57 binds to oJM39 with even higher affinity than that observed with oNP42, an ORF57-binding RNA oligo from KSHV vIL-6 MRE used as a positive control [18] (Figure 2D). All remaining RNA oligos from other parts of the ORF59 MRE showed no binding affinity to ORF57 similarly to a negative control RNA oligo oNP41. These data indicate the importance of loop sequences of the ORF59 MRE for ORF57 binding, similar to the MREs indentified in vIL-6 and PAN [18,19].

Our previous studies have shown that ORF57 affects ORF59 accumulation by interacting with RBM15, an RNA binding export cofactor [15,29]. Therefore, we next tested the interaction of ORF59 MRE with RBM15 using the same set of RNA oligos. We found that, similarly to ORF57, RBM15 binds oJM39 and oNP42 (a positive control), but only very weakly to other ORF59 MRE RNA oligos (Figure 2D). RBM15 binding to oJM39 and oNP42 was independent of ORF57 even in the cell extract (V) without ORF57 expression from the TREx BCBL1-vector cells. The reduced RBM15 interaction with oJM39 and oNP42 in the extract from TREx BCBL1-RTA cells (R) than in the TREx BCBL1-vector cell extract (V) (Figure 2D) was caused by the reduced expression of RBM15 in TREx BCBL1–RTA cells (compare the input RBM15 of R *vs*. V in Figure 2D).

**Figure 3:**
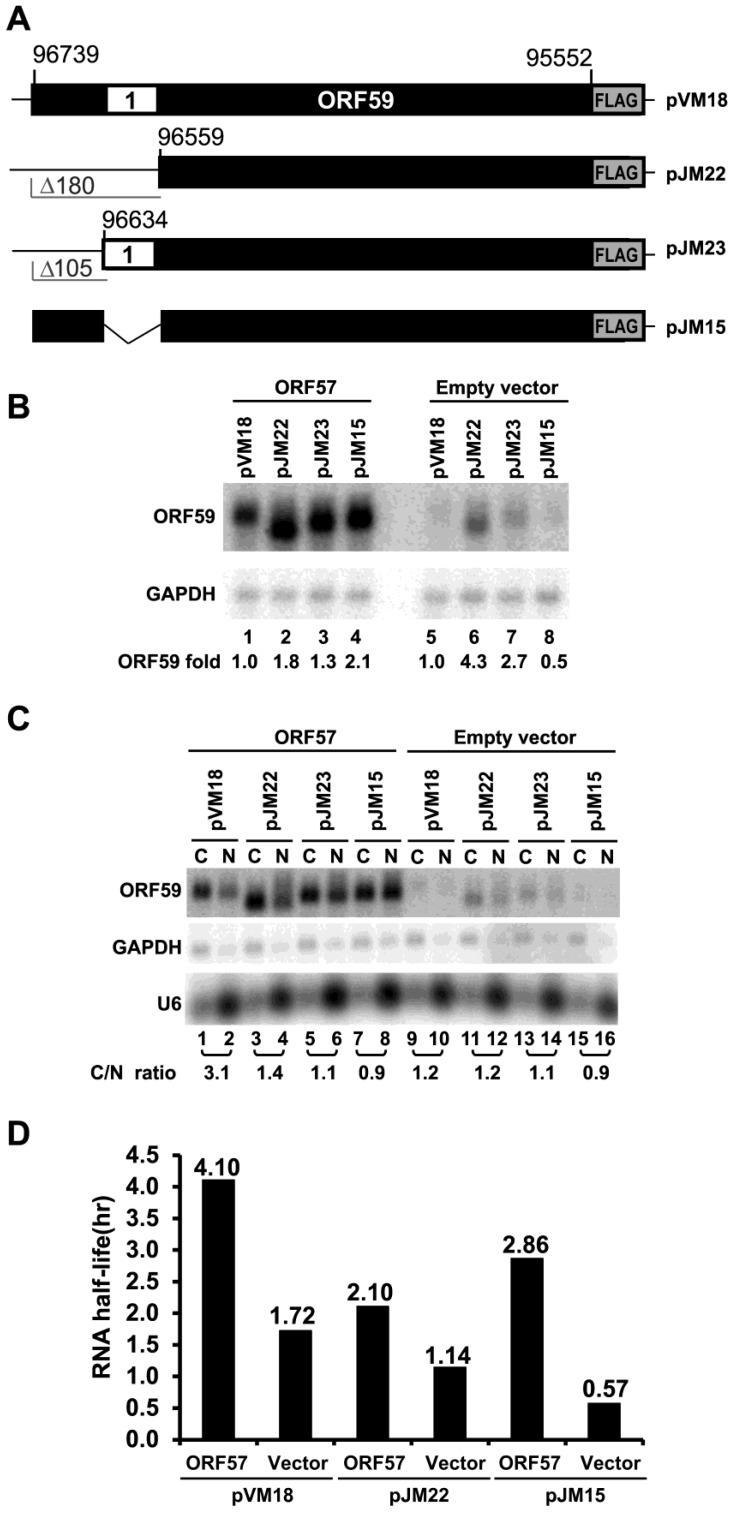
The role of 5’ ORF59 MRE in ORF59 RNA export and half-life. (**A**) Diagrams of wt ORF59 and its 5’ in-frame deletion mutants. See other details in Figure 1. (**B**) Expression of ORF59 RNA in HEK293 cells. Total RNA from HEK293 cells transfected with individual ORF59 construct in the presence or absence (empty vector) of ORF57-FLAG was examined by Northern blot with a ^32^P-labeled oligo probe (oVM158) for ORF59 RNA and oZMZ270 for GAPDH used as a loading control. A relative level of ORF59 RNA in each sample after normalization to the corresponding GAPDH level is shown at the bottom of Northern blots, with the level of wt ORF59 RNA being set to 1. (**C**) Examination of the 5’ MRE in export of ORF59 RNA. Fractionated cytoplasmic (C) or nuclear (N) RNA from HEK293 cells cotransfected with wt ORF59 (pVM18) and its MRE deletion mutants (pJM22, pJM23 and pJM15) was examined by Northern blot as described in (**B**) for ORF59 RNA. GAPDH RNA and U6 RNA served as fractionation efficiency controls. C/N ratio was calculated based on relative level of ORF59 in the cytoplasm *versus* the nucleus. (**D**) Half-life of wt and mt ORF59 RNA in the presence of ORF57 or an empty vector. HEK293 were transfected with a vector expressing wt or mt ORF59 together with an ORF57 expression vector or an empty vector control. At 24 h of transfection, RNA transcription was stopped by addition of 10 µg/mL of actinomycin D and total RNA was extracted from each sample over the time (hr) for the remaining ORF59 and GAPDH (for normalization) RNA quantified by qRT-PCR. Results are presented as calculated half-life for each study group in three separate experimental repeats. Holm’s method adjusted *p*-values for pair-wise comparisons between the corresponding estimated decay rate parameters as shown before [15,19] are presented in detail in Table S3.

To evaluate the relevance and functionality of the ORF59 5’ MRE sequence, we analyzed the MRE truncation mutants pJM22 and pJM23 (Figure 1A) further and constructed a mutant with an internal deletion of the MRE sequence (pJM15) (Figure 3A). These mutants were evaluated for their response to ORF57 in co-transfection experiments in HEK293 cells. Figure 3B shows that all three mutants exhibited increased RNA accumulation, compared to wt ORF59 RNA expressed from pVM18 in the presence of ORF57 (Figure 3B). However, the background RNA expression of all three ORF59 mutants was different from pVM18 wt ORF59 RNA (Figure 3B, Lanes 5–8). All three mutants also exhibited decreased efficiency of RNA export, in particular the nuclear export of pJM15 ORF59 RNA (Figure 3C) in the presence of ORF57, with a lower C/N ratio over that of the wt ORF59 RNA (pVM18). It remains to know why the mutant ORF59 RNA expressed from pJM23 with a 5’ MRE displayed a similar pattern to the one expressed from pJM15 without a MRE. In addition, we found that the mutant ORF59 RNA expressed from pJM22 with a larger 5’ deletion than pJM15 was less stable than wt ORF59 expressed from pVM18 in the presence of ORF57, whereas the mutant ORF59 RNA expressed from pJM15 was less stable than wt ORF59 RNA expressed from pVM18 in the absence of ORF57, with a half-life shorter than wt ORF59 (Figure 3D, Table S3). Insertion of the MRE alone or together with its upstream 105 nts into the 5’ GFP displayed no effect on GFP expression (data not shown), suggesting that the MRE identified in the ORF59 has to work with other uncharacterized element(s) in ORF59 RNA for its function.

### 3.3. ORF59 RNA without the 5' MRE is Translatable in the Presence of ORF57, but Poorly Translatable in the Presence of RBM15

**Figure 4:**
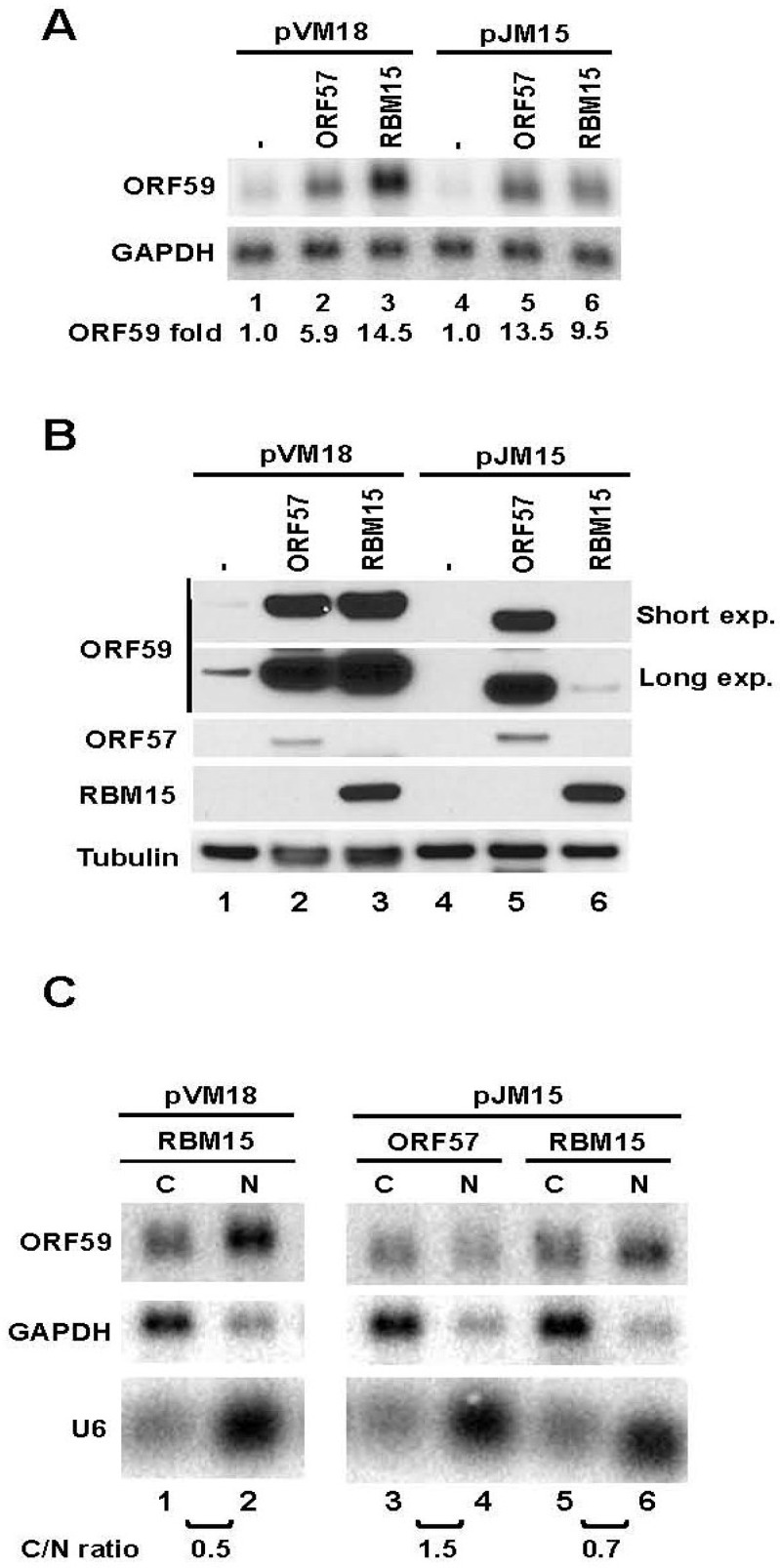
ORF57 but not RBM15 promotes protein translation from an ORF59 MRE-deletion mutant. (**A**) and (**B**) Total RNA from HEK293 cells transfected with each of the ORF59 constructs in the absence or presence of ORF57, or RBM15 was examined by Northern blot (**A**) as described in Figure 1B for the expression of ORF59 and GAPDH. Protein samples from the same set of HEK293 cells were examined by Western blot (**B**) using an anti-FLAG antibody to detect the expression of both ORF59 and ORF57 or an anti- β-tubulin antibody for sample loading. Two exposures (exp.), short and long, are shown for anti-FLAG ORF59 blot. (**C**) Fractionated cytoplasmic (C) or nuclear (N) RNA from HEK293 cells transfected with wt ORF59 (pVM18) or its MRE-deletion mutant (pJM15) in the presence of ectopic expression of RBM15 or ORF57 was examined by Northern blot as described in Figure 3C for ORF59 RNA expression and calculated as a C/N ratio for its cytoplasmic *versus* nuclear RNA levels. Both GAPDH and U6 RNAs were blotted for fractionation efficiency.

KSHV ORF57 interacts with RNA export factor Aly/REF and NXF1 [20,30] and cofactors RBM15 and OTT3 by protein-protein interaction [15]. ORF57 affects the RNA-binding activity of RBM15 and prevents RBM15-mediated nuclear accumulation of hyperpolyadenylated ORF59 RNA [15]. Accordingly, we tested whether the ORF59 mutant in pJM15 carrying an internal deletion of the ORF59 MRE responds to RBM15 upon cotransfection of HEK293 cells (Figure 4A). As expected, ORF57 enhanced the expression of both wt ORF59 in pVM18 and mutant ORF59 in pJM15 both at their total RNA and protein levels. To our surprise, RBM15 cotransfection led to enhanced expression of both wt and mt RNAs, but only to increased protein expression of wt ORF59 in pVM18, not the mutant ORF59 in pJM15 (Figure 4A,B). Analysis of the relative C/N ratio in RNA fractionation experiments showed that ectopic RBM15 led to more nuclear RNA of both wt and mutant ORF59, indicating that RBM15 promotes the nuclear accumulation independently of the 5' MRE (Figure 4C). Thus, nuclear accumulation of the mt ORF59 RNA mediated by RBM15 would not be a plausible explanation for the lack of protein expression from the mt ORF59 RNA. Together with the data from Figure 3, our results suggest that the identified 5’ MRE motif and its surrounding region in ORF59 RNA are more likely involved in the export of ORF59 RNA and are responsible for RBM15-mediated protein translation.

**Figure 5:**
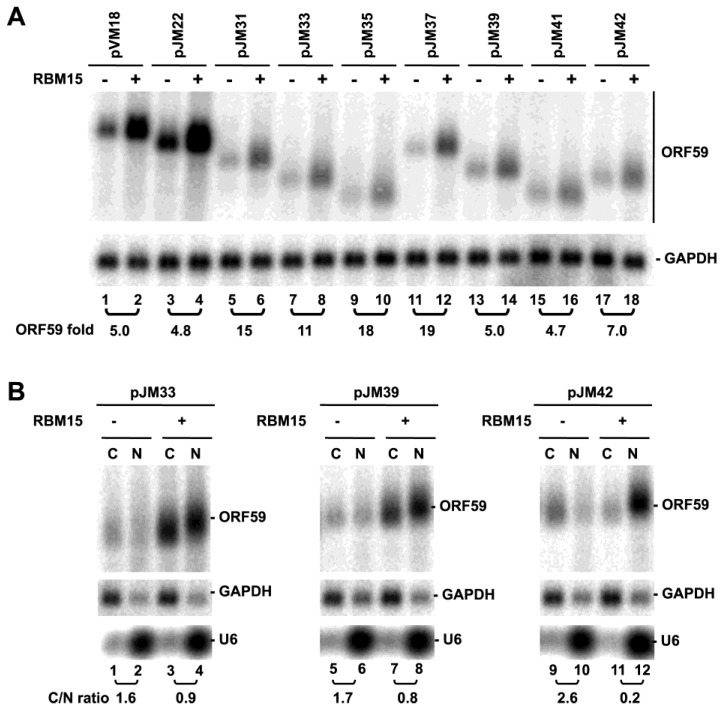
RBM15-mediated RNA accumulation of wt and mt ORF59 in the absence of ORF57. (**A**) Total RNA from HEK293 cells transfected with each of ORF59 constructs described in Figure 1A in the presence (+) or absence (−) of RBM15 were analyzed by Northern blot analysis as described in Figure 1B for ORF59 RNA expression using antisense oligo probe oVM158 for pVM18, pJM22, pJM31, pJM33 and pJM35 or antisense oligo probe oJM65 for pJM37, pJM39, pJM41 and pJM42. The same membrane was re-probed separately with GAPDH-specific probe for sample loading. (**B**) RBM15 promotes ORF59 expression and nuclear accumulation of the hyperpolyadenylated ORF59 RNA. Fractionated cytoplasmic (C) and nuclear (N) RNA from HEK293 cells cotransfected with each ORF59 construct in the presence (+) or absence (−) of RBM15 was examined by Northern blot for ORF59 RNA expression as a C/N ratio for its cytoplasmic *versus* nuclear RNA levels as described above. GAPDH RNA and U6 RNA served for fractionation efficiency.

We also analyzed RBM15's ability to accumulate RNA from other progressive 3’- and 5’-truncation mutants of ORF59. All of the mutants analyzed showed higher levels of ORF59 RNA expression in the presence of RBM15 (Figure 5A). In agreement with our previous study, RBM15-enhanced ORF59 RNA migrated slower and appeared with smear when compared with the RNA in the absence of RBM15 (Figure 5A). Nuclear and cytoplasmic fractionation analysis indicated that three selected ORF59 mutants exhibited RNA accumulation in the nuclear fraction in the presence of RBM15 and slower migration than its counterpart in the cytoplasmic fraction in electrophoresis (Figure 5B), indicating that the accumulated nuclear ORF59 RNA is of a larger size due to hyperpolyadenylation [15] regardless of where the truncation was made. Although the observed RNA hyperpolyadenylation is preventable in the presence of ORF57 as we reported for the wt ORF59 RNA [15], further experiments are needed to confirm the prediction.

## 4. Conclusions

In summary, this study has demonstrated that KSHV ORF59 contains multiple regions or motifs responsible for viral ORF57 and cellular RBM15. ORF57 promotes protein translation from ORF59 RNA and requires two terminal regions or one terminal region plus a central region of ORF59 for this function. The MRE identified by anti-ORF57 CLIP binds ORF57 protein and RBM15 and appears to be important for RNA export and stability of ORF59. However, this MRE motif is also important for RBM15-mediated protein translation of ORF59. Together, these studies provide further insight into how ORF57 promotes ORF59 expression by balanced interplay with RBM15.

## Supplementary Files

Supplementary File 1
