# Supplementary material for: Multiple Regions of Kaposi’s Sarcoma-Associated Herpesvirus ORF59 RNA are Required for Its Expression Mediated by Viral ORF57 and Cellular RBM15
Source: Viruses. 2015 Feb 3;7(2):496–510. doi: 10.3390/v7020496 (PMC4353900; doi:10.3390/v7020496)
Supplement: Supplementary File 1 [file viruses-07-00496-s001.pdf]

Supplementary Tables

Table S1. Plasmids used in this study. Characteristics include antibiotics resistance (Amp^r, ampicillin resistant), parent vectors, cloned inserts and brief cloning strategies. DNA oligo name and its nt position in KSHV genome (GenBank Acc. No. U75698.1) employed for individual plasmid construction are provided in Table S2.

Plasmid	Characteristics	Oligos Used to Amplify the Insert
**For ORF59 expression**		
pJM15	Ap^r; pFLAG-CMV-5.1 (Sigma) with a EcoRI-BamHI insert corresponding to ORF59 with an internal deletion of ORF59 MRE element	oJM30, oJM31, oVM88 and oVM89
pJM22	Ap^r; pFLAG-CMV-5.1 (Sigma) with a EcoRI-BamHI insert corresponding to ORF59 with a 5' deletion of 180 nt	oJM44 and oVM88
pJM23	Ap^r; pFLAG-CMV-5.1 (Sigma) with a EcoRI-BamHI insert corresponding to ORF59 with a 5'deletion of 105 nt	oJM45 and oVM88
pJM30	Ap^r; pFLAG-CMV-5.1 (Sigma) with a EcoRI-BamHI insert corresponding to ORF59 with a 5' deletion of 312 nt	oJM54 and oVM88
pJM31	Ap^r; pFLAG-CMV-5.1 (Sigma) with a EcoRI-BamHI insert corresponding to ORF59 with a 5' deletion of 430 nt	oJM55 and oVM88 (
pJM32	Ap^r; pFLAG-CMV-5.1 (Sigma) with a EcoRI-BamHI insert corresponding to ORF59 with a 5' deletion of 560 nt	oJM56 and oVM88
pJM33	Ap^r; pFLAG-CMV-5.1 (Sigma) with a EcoRI-BamHI insert corresponding to ORF59 with a 5' deletion of 670 nt	oJM57 and oVM88
pJM34	Ap^r; pFLAG-CMV-5.1 (Sigma) with a EcoRI-BamHI insert corresponding to ORF59 with a 5' deletion of 768 nt	oJM58 and oVM88
pJM35	Ap^r; pFLAG-CMV-5.1 (Sigma) with a EcoRI-BamHI insert corresponding to ORF59 with a 5' deletion of 858 nt	oJM59 and oVM88
pJM36	Ap^r; pFLAG-CMV-5.1 (Sigma) with a EcoRI-BamHI insert corresponding to ORF59 with a 3' deletion of 123 nt	oVM89 and oJM60
pJM37	Ap^r; pFLAG-CMV-5.1 (Sigma) with a EcoRI-BamHI insert corresponding to ORF59 with a 3' deletion of 270 nt	oVM89 and oJM61
pJM38	Ap^r; pFLAG-CMV-5.1 (Sigma) with a EcoRI-BamHI insert corresponding to ORF59 with a 3' deletion of 435 nt	oVM89 and oJM62
pJM39	Ap^r; pFLAG-CMV-5.1 (Sigma) with a EcoRI-BamHI insert corresponding to ORF59 with a 3' deletion of 555 nt	oVM89 and oJM63
pJM40	Ap^r; pFLAG-CMV-5.1 (Sigma) with a EcoRI-BamHI insert corresponding to ORF59 with a 3' deletion of 681 nt	oVM89 and oJM64
pJM41	Ap^r; pFLAG-CMV-5.1 (Sigma) with a EcoRI-BamHI insert corresponding to ORF59 with a 3' deletion of 804 nt	oVM89 and oJM65
pJM42	Ap^r; pFLAG-CMV-5.1 (Sigma) with a EcoRI-BamHI insert corresponding to ORF59 with a 5' deletion of 312 nt and a 3' deletion of 270 nt.	oJM54 and oJM61

Table S2. DNA and RNA oligos used in this study.

Oligo Name	Position	Features *	Sequence
oVM88	KSHV ORF59 nt 95552-72	BamHI, F	5'-ACTCAGGATCC/AATCAGGGGGTTAAATGTGGT-3'
oVM89	KSHV ORF59 nt 96719-39	EcoRI, B	5'-TACTAGAATTCACC/ATGCCTGTGGATTTTCACTAT-3'
oJM30	KSHV ORF59 nt 96540-58/96634-52	F	5'-GTAAGGGCATTCTTTATTC/GCACTACTCCGGTTTTAGT-3'
oJM31	KSHV ORF59 nt 96652-34/96558-40	B	5'-ACTAAAACCGGAGTAGTGC/GAATAAAGAATGCCCTTAC-3'
oJM36	KSHV ORF59 nt 96633-09	B	Biotin-AAGUGCACGGAUCGGCUUGCACGCC
oJM37	KSHV ORF59 nt 96608-584	B	Biotin-AACCCUCAGUGUGCUGUCCAGCGUG
oJM38	KSHV ORF59 nt 96583-59	B	Biotin-GGGACAGCUGGCGUUCUGGGGUUAA
oJM39	KSHV ORF59 nt 96596-72	B	Biotin-GCUGUCCAGCGUGGGGACAGCUGGC
oJM44	KSHV ORF59 nt 96559-39	EcoRI, B	5'-TACTAGAATTCACC/ATG/AGAATAAAGAATGCCCTTACG-3'
oJM45	KSHV ORF59 nt 96634-17	EcoRI, B	5'-TACTAGAATTCACC/ATG/CAAGTGCACGGATCGGCT-3'
oJM54	KSHV ORF59 nt 96430-12	EcoRI, B	5'-TACTAGAATTCACC/ATG/CTCGACGCTGGCATAGCCT-3'
oJM55	KSHV ORF59 nt 96310-292	EcoRI, B	5'-TACTAGAATTCACC/ATG/GTGGTGGACCAAAAGGGTA-3'
oJM56	KSHV ORF59 nt 96181-62	EcoRI, B	5'-TACTAGAATTCACC/ATG/GAGGACCAAACGGTGACTGT-3'
oJM57	KSHV ORF59 nt 96070-53	EcoRI, B	5'-TACTAGAATTCACC/ATG/GGCTGGGATGGTCTGAAG-3'
oJM58	KSHV ORF59 nt 95974-55	EcoRI, B	5'-TACTAGAATTCACC/ATG/CTGAGACTGTGTAAAGTCCC-3'
oJM59	KSHV ORF59 nt 95884-66	EcoRI, B	5'-TACTAGAATTCACC/ATG/TGCCAATCAGGTGACGTAA-3'
oJM60	KSHV ORF59 nt 95672-89	BamHI, F	5'-ACTCAGGATCC/GGTGACAGAGGCAGCGTC-3'
oJM61	KSHV ORF59 nt 95819-36	BamHI, F	5'-ACTCAGGATCC/CTCGGCAGACACAGATCG-3'
oJM62	KSHV ORF59 nt 95984-6002	BamHI, F	5'-ACTCAGGATCC/TAAGGACTCCAGGCTAACC-3'
oJM63	KSHV ORF59 nt 96104-22	BamHI, F	5'-ACTCAGGATCC/GATCTTGCTCACGCCACCA-3'
oJM64	KSHV ORF59 nt 96230-47	BamHI, F	5'-ACTCAGGATCC/GCCACAGAGGAGAAGCCT-3'
oJM65	KSHV ORF59 nt 96353-71	BamHI, F	5'-ACTCAGGATCC/GGTGGTCCTGACGAACTGC-3'
oNP41	KSHV vIL6 nt 17498-76	B	Biotin-GCUUCUGACGAAGACCUUAGGAU
oNP42	KSHV vIL6 nt 17483-59	B	Biotin-CUUAGGAUGGGACAUACAGGAAGAG
oZMZ296	pEGFP-C1 nt 880-62	B	5'-GCATGGCGGACTTGAAGAA-3'
oVM158	KSHV ORF59 nt 95717-37	F	5'-GGGACCAACTGGTGTGAGAGG-3'
oZMZ243	T7 Promoter, B strand	B	5'-CTATAGTGAGTCGTATTAAT-3'
oVM11	KSHV ORF57 nt 82296-77	B	5'-CTCGTCTTCCAGTGTCGGTG-3'
ORF59-P	KSHV ORF59 Probe	F, TaqMan	5'-/56-FAM/AAACCGATCTGTGTCTGCCGAGG/3IABkFG/-3'
ORF59-1	KSHV ORF59 Primer 1	F, TaqMan	5'-TTAGAAGTGGAAGGTGTGCC-3'
ORF59-2	KSHV ORF59 Primer 2	B, TaqMan	5'-TCCTGGAGTCCGGTATAGAATC-3'
oZMZ270	Human GAPDH, NM_002046	B	5'-TGAGTCCTTCCACGATACCAAA-3'
oST197	Human U6	B	5'-AAAATATGGAACGCTTCACGA-3'

* F, forward; B, backward.

Table S3. Half-life calculation of wt ORF59 pVM18 and mt ORF59 oJM22 and oJM15 lacking the 5' MRE in the presence or absence of ORF57. Experiments in three repeats were performed as described in Figure 3D using non-linear regression analysis as shown before [15,19]. Briefly, a non-linear regression analysis on the raw data was performed, choosing an exponential decay model [Fold Percent = α*exp (β*time)] where α (alpha) is the intercept when time = 0, and β (beta) is the decay rate. (**A**) Non-linear parameter (b) and half-life estimates (a = 100% ± 1%) present the estimated betas (Est. b), their standard errors (SE), and the two-tailed p-values for the test that the estimated betas were equal to zero according to treatment and where alpha (a) was constrained to be equal to 100%. Also shown is the adjusted R-squared (all of which indicate a satisfactory fit) and predicted half-life (hrs) for each of the treatments. (**B**) Treatment comparison results present the differences between the estimates betas, their standard errors, and the two-tailed adjusted p-values for the indicated pair-wise comparisons between the estimated betas. All but four of the comparisons were significantly different.

A. Non-linear parameter (b) and half-life estimates (a=100% ± 1%).

Treatment	Est. b	SE	P	Adj. R-sq.	time (1/2)
pVM18 + Empty vector	−0.403	0.0375	<0.0001	0.87	1.72
pVM18+ORF57	−0.169	0.0321	<0.0001	0.94	4.10
pJM22 + Empty vector	−0.608	0.106	0.0001	0.65	1.14
pJM22+ORF57	−0.330	0.0385	<0.0001	0.92	2.10
pJM15 + Empty vector	−1.21	0.173	0.0001	0.72	0.57
pJM15+ORF57	−0.242	0.0424	0.0005	0.95	2.86

B. Treatment comparison results.

Comparison	Estimated Difference *	SE	P**
pVM18 + Empty vector *vs*. pVM18 + ORF57	0.236	0.0446	<0.0001
pJM22 + Empty vector *vs*. pJM22 + ORF57	0.278	0.107	0.067
pVM18 + ORF57 *vs*. pJM22 + ORF57	0.163	0.0428	0.005
pVM18 + Empty vector *vs*. pJM22 + ORF57	0.0727	0.0543	0.22
pVM18 + ORF57 *vs*. pVM18 + Empty vector	0.441	0.0958	0.0008
pVM18 + Empty vector *vs*. pJM22 + Empty vector	0.205	0.0990	0.14
pJM15 + Empty vector *vs*. pJM15 + ORF57	0.986	0.233	0.005
pVM18 + ORF57 *vs*. pJM15 + ORF57	0.0706	0.0423	0.22
pVM18 + Empty vector *vs*. pJM15 + ORF57	0.169	0.0569	0.033
pVM18 + ORF57 *vs*. pJM15 + Empty vector	1.04	0.219	0.0008
pVM18 + Empty vector *vs*. pJM15 + Empty vector	0.806	0.186	0.002

* Absolute estimated difference between beta's; ** Adjusted p-values using Holm's method.
